# Supplementary material for: Observation of domain wall bimerons in chiral magnets
Source: Nat Commun. 2021 Jun 9;12:3490. doi: 10.1038/s41467-021-23845-y (PMC8190141; doi:10.1038/s41467-021-23845-y)
Supplement: Supplementary file 1 — Supplementary Information [file 41467_2021_23845_MOESM1_ESM.pdf]

Supplementary Information for
"Observation of domain wall bimerons in chiral magnets"

T. Nagase[1*], Y. G. So[2], H. Yasui[2], T. Ishida[3,4], H. K. Yoshida[5], Y. Tanaka[4], K. Saitoh[3,4], N. Ikarashi[1,6], Y. Kawaguchi[4], M. Kuwahara[3,4*] and M. Nagao[1,6*]

[1]Department of Electronics, Graduate School of Engineering, Nagoya University, Nagoya 464-8603, Japan.

[2]Department of Materials Science, Graduate School of Engineering Science, Akita University, Akita 010-8502, Japan.

[3]Advanced Measurement Technology Center, Institute of Materials and Systems for Sustainability, Nagoya University, Nagoya 464-8601, Japan.

[4]Department of Applied Physics, Graduate School of Engineering, Nagoya University, Nagoya 464-8603, Japan.

[5]Department of Physics, Faculty of Science, Hokkaido University, Sapporo 060-0810, Japan.

[6]Center for Integrated Research of Future Electronics, Institute of Materials and Systems for Sustainability, Nagoya University, Nagoya 464-8601, Japan.

*e-mail: nagase.tomoki@k.nagoya-u.jp; kuwahara@imass.nagoya-u.ac.jp; nagao.masahiro@imass.nagoya-u.ac.jp.

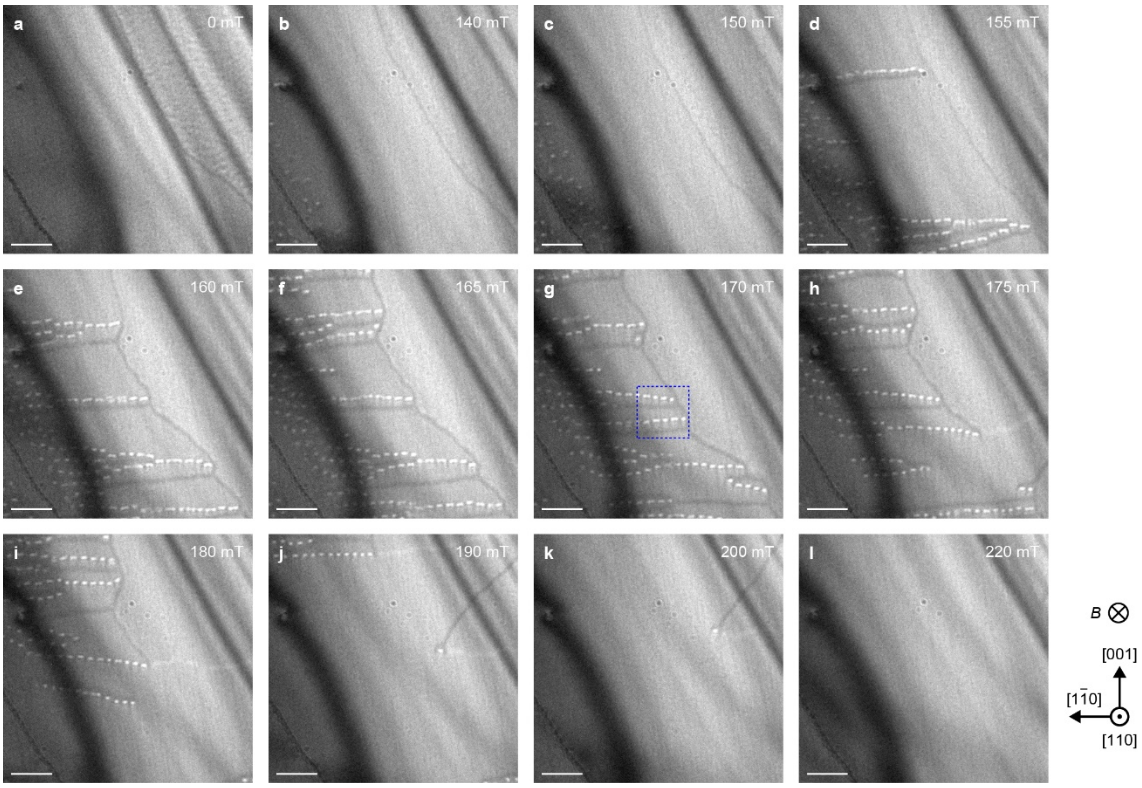

Supplementary Fig. 1 | Detail of evolution of magnetic domain structures in the $Co_{8.5}Zn_{7.5}Mn_4$(110) thin-film with t ~ 50 nm. A series of underfocused ($\Delta f = -2$ mm) LTEM images obtained at 330 K. The scale bars are 1 μm. The blue dotted box in **g** corresponds to Fig. 3a-c of the main text.

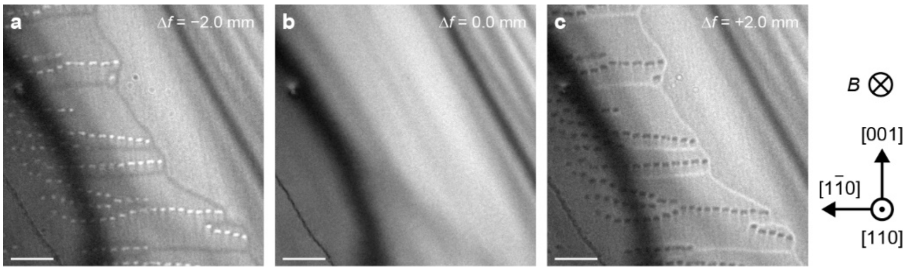

Supplementary Fig. 2 | LTEM images with various defocus values. Underfocused (Δ*f* = −2 mm) (**a**), in-focus (Δ*f* = 0 mm) (**b**) and overfocused (Δ*f* = +2 mm) (**c**) LTEM images obtained at 330 K and 170 mT corresponding to Figs. 2d and S1g. The scale bars are 1 μm. The bright and dark contrasts originating from magnetic flux reverse for **a** and **c**. There is no magnetic contrast in **b**. The strong dark line contrasts (the so-called bend contours) seen in the upper left to lower middle of each figure appear at locations where the Bragg condition is locally satisfied due to sample bending.

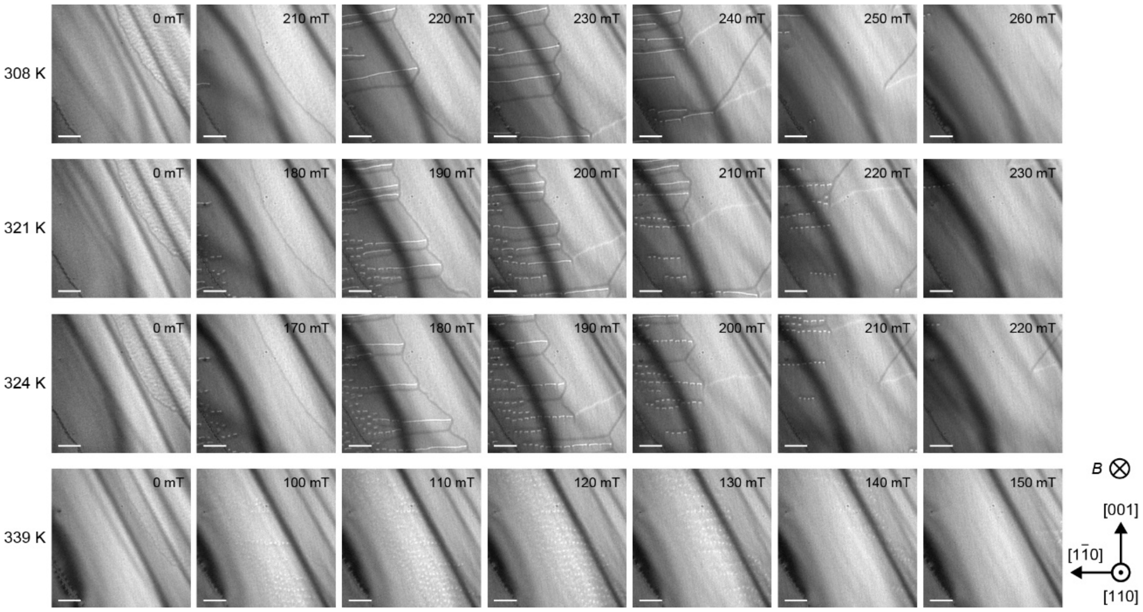

Supplementary Fig. 3 | DW bimerons at various temperatures. Underfocused ($\Delta f = -2$ mm) LTEM images obtained in the $Co_{8.5}Zn_{7.5}Mn_4$(110) thin-film with t ~ 50 nm at various temperatures and magnetic fields. The scale bars are 1 μm.

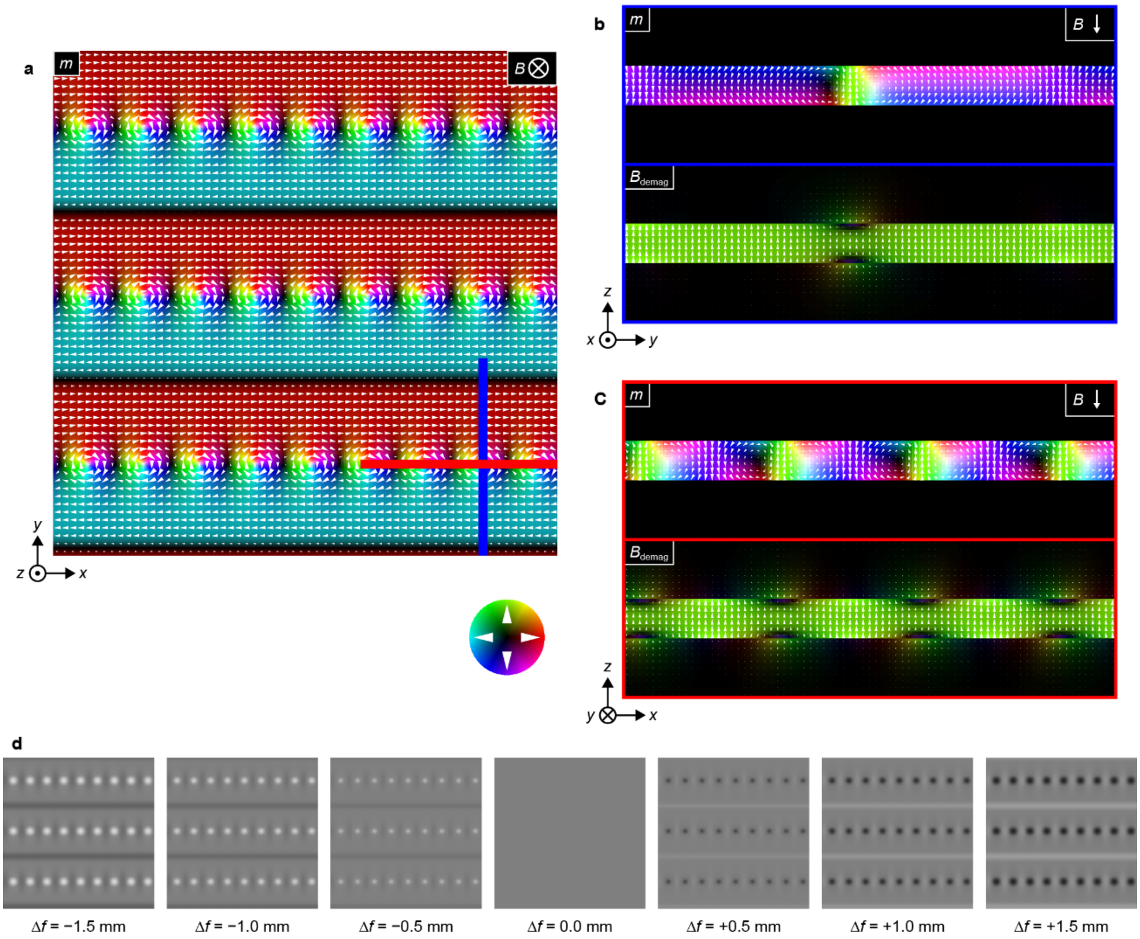

Supplementary Fig. 4 | Detail of micromagnetic and LTEM image simulations of DW bimerons in a cubic chiral magnet (110) thin-film with the magnetic easy axes of the <100> directions (K_c > 0). The parameters are $K_c = +K_0$ and $B = 2.75B_d$ corresponding to Fig. 4b in the main text. The thickness is 50 nm. **a**, The simulated magnetic structure of DW bimerons in the *xy*-plane. The size of the image is 1.6×1.6 μm^2. **b**,**c**, The simulated magnetic structure (upper panel) and the demagnetization field (lower panel) of the DW bimerons in the *yz*-plane (**b**) and *xz*-plane (**c**). **b** and **c** are cross-sections corresponding to the blue and red lines in **a**, respectively. The colour and arrows represent the orientation and magnitude of the in-plane component of the magnetization and demagnetization field. White and black indicate upward and downward directions, respectively, relative to the paper. **b** shows that the magnetic domains have conical spin structures. **d**, A series of simulated LTEM images of the DW bimerons. The leftmost and rightmost panels are the same as Fig. 4d,e of the main text, respectively.

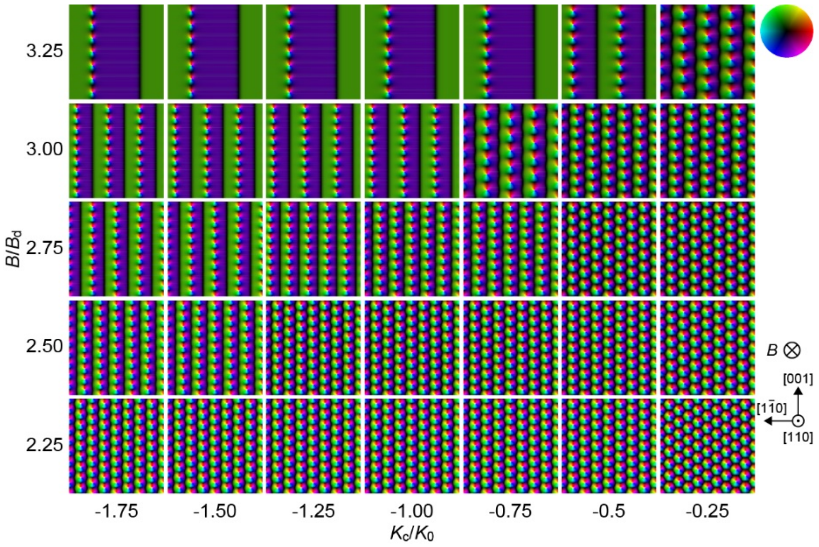

Supplementary Fig. 5 | Micromagnetic simulation in (110) thin-films of cubic chiral magnets with the magnetic easy axes of the <111> directions (K_c < 0). Simulated results of magnetization distributions for various cubic magnetocrystalline anisotropy (K_c) and magnetic fields (B). The size of each image is 1.6×1.6 μm^2 and the thickness is t = 50 nm. The colour wheel (upper right panel) represents the orientation and magnitude of the in-plane magnetization. White and black indicate $+z$ and $-z$ directions, respectively.

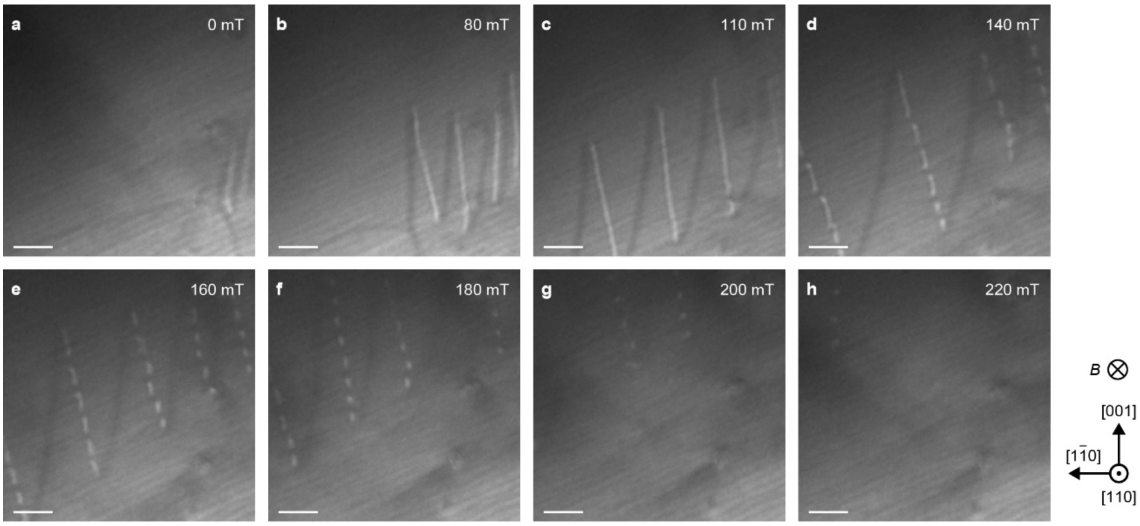

Supplementary Fig. 6 | DW bimerons in the $Co_7Zn_{11}Mn_2$(110) thin-film. A series of underfocused LTEM images ($\Delta f = -2$ mm) obtained at 401 K in a (110) thin-film of $Co_7Zn_{11}Mn_2$ which has Curie temperature of ~410 K and helical spin period of ~180 nm. The scale bars are 500 nm. Conventional DWs along the <100> directions are observed (**a-c**). With increasing magnetic field, the DWs with the bright line alter to the DW bimerons along the <100> directions (**d-f**).
